# The clinical significance of snail protein expression in gastric cancer: a meta-analysis

**DOI:** 10.1186/s40246-016-0070-6

**Published:** 2016-07-25

**Authors:** Xiaoya Chen, Jinjun Li, Ling Hu, William Yang, Lili Lu, Hongyan Jin, Zexiong Wei, Jack Y. Yang, Hamid R. Arabnia, Jun S. Liu, Mary Qu Yang, Youping Deng

**Affiliations:** 1 Medical College, Wuhan University of Science and Technology, Wuhan, 430065 China; 2 Department of Anesthesiology, Tianyou Hospital, Wuhan University of Science and Technology, Wuhan, 430064 China; 3 Texas Advanced Computing Center, University of Texas at Austin, 10100 Burnet Road, Austin, TX 78758 USA; 4 Puren Hospital, Wuhan University of Science and Technology, Wuhan, 430081 China; 5 MidSouth Bioinformatics Center, Department of Information Science, George Washington Donaghey College of Engineering and Information Technology and Joint Bioinformatics Graduate Program, University of Arkansas at Little Rock and University of Arkansas for Medical Sciences, 2801 S. University Avenue, Little Rock, AR 72204 USA; 6 Department of Statistics and Harvard School of Public Health, Harvard University, One Oxford St., Cambridge, 02138 Massachusetts USA; 7 Department of Computer Science, University of Georgia, Athens, GA 30602 USA; 8 Department of Internal Medicine and Rush University Cancer Center, Rush University Medical Center, Chicago, IL 60612 USA

**Keywords:** Gastric cancer, Snail, Meta-analysis

## Abstract

**Background:**

Snail is a typical transcription factor that could induce epithelial-mesenchymal transition (EMT) and cancer progression. There are some related reports about the clinical significance of snail protein expression in gastric cancer. However, the published results were not completely consistent. This study was aimed to investigate snail expression and clinical significance in gastric cancer.

**Results:**

A systematic review of PubMed, CNKI, Weipu, and Wanfang database before March 2015 was conducted. We established an inclusion criterion according to subjects, method of detection, and results evaluation of snail protein. Meta-analysis was conducted using RevMan4.2 software. And merged odds ratio (OR) and 95 % CI (95 % confidence interval) were calculated. Also, forest plots and funnel plot were used to assess the potential of publication bias.

A total of 10 studies were recruited. The meta-analysis was conducted to evaluate the positive rate of snail protein expression. OR and 95 % CI for different groups were listed below: (1) gastric cancer and para-carcinoma tissue [OR = 6.15, 95 % CI (4.70, 8.05)]; (2) gastric cancer and normal gastric tissue [OR = 17.00, 95 % CI (10.08, 28.67)]; (3) non-lymph node metastasis and lymph node metastasis [OR = 0.40, 95 % CI (0.18, 0.93)]; (4) poor differentiated cancer, highly differentiated cancer, and moderate cancer [OR = 3.34, 95 % CI (2.22, 5.03)]; (5) clinical stage TI + TII and stage TIII + TIV [OR = 0.38, 95 % CI (0.23, 0.60)]; (6) superficial muscularis and deep muscularis [OR = 0.18, 95 % CI (0.11, 0.31)].

**Conclusions:**

Our results indicated that the increase of snail protein expression may play an important role in the carcinogenesis, progression, and metastasis of gastric cancer. And this result might provide instruction for the diagnosis, therapy, and prognosis of gastric cancer.

## Background

Epithelial-mesenchymal transition (EMT), a developmental process whereby epithelial cells reduce intercellular adhesion and acquire myofibroblastic features, is critical to tumor progression [1–3]. Meanwhile, the dissolution of intercellular adhesions and the acquisition of a more motile mesenchymal phenotype as part of epithelial-to-mesenchymal transition (EMT) are crucial capacities of invading cancer cells [4]. Snail can induce EMT partly by suppressing the expression of E-cadherin. Reduced expression of E-cadherin may lead to the loss of cell-cell adhesion and cancer progression [5]. In recent years, snail was found to be highly expressed in several carcinomas, including non-small cell lung carcinomas, ovarian carcinomas, urothelial carcinomas, breast cancer, and hepatocellular carcinoma [6–10]. Studies of immunohistochemical analyses suggest that snail is highly expressed in gastric cancer and significantly associated with tumor progression and metastasis [11–13].

## Methods

### Study search protocol

A total of 10 studies were identified by primary search strategies using the keywords “snail” combined with “gastric cancer” and synonyms in PubMed, CNKI, Weipu, and Wanfang database.

### Inclusion criteria and exclusion criteria

Studies that were included in this meta-analysis met the following criteria: (1) the official published literature or master’s and doctoral dissertation in both Chinese and English before March 2015; (2) the detection method used immunohistochemical and the results experienced quantitative analysis; (3) when duplicate articles were published, we included the newest or the most informative single study; (5) the snail positive rate was given or could be calculated based on the information from tables or figures.

Exclusion criteria included (1) repetitive studies; (2) research on animal and cellular level; (3) studies without reviews, letters, abstracts and editorials; and (4) the studies without control group.

### Data extraction and quality assessment

Two reviewers screened the titles and abstracts according to the inclusion and exclusion criteria listed above independently. Then, they cross-checked the articles and removed disagreements. Information extracted from the eligible articles included first author, publication year, detection method, the number of cases and controls, the clinical pathology states of cases and controls, and the location of snail protein expression. The quality of these studies is assessed by the following: (1) whether the gold standard method is set up; (2) whether the gold standard test stayed is independent of the evaluation test; (3) whether the blind method is used; (4) whether quantitative data is given or is able to be calculated; (5) whether the definition and diagnosis of the case are correct, independent, and standard; (6) whether the diagnostic steps are detailed; (7) whether the case has a good representation; (8) whether cases and controls are selected and analyzed based on the most important factor. Based on the above standards, we classified the qualities of the research into five grades: (A) meets all 8 quality standards; (B) meets 7 standards; (C) meets 6 standards; (D) meets 5 standards; (E) meets 4 standards.

### Statistical analysis

Meta-analysis was conducted with RevMan4.2 software. Odds ratio (OR) with 95 % confidence interval was calculated. Heterogeneity between studies was examined using the I2 statistic [14, 15]. When I2 value was greater than 50 %, we considered that heterogeneity was significant. Fixed-effect Mantel-Haenszel model was chosen as the main analysis method when the heterogeneities were not confirmed statistically significant. Otherwise, random-effect model was adopted. Funnel plots were used to check for the potential of publication bias. All the *P* values were two-sided, and statistically significant difference was defined as *P* < 0.05.

## Results

### Literature search and study characteristics

**Fig. 1:**
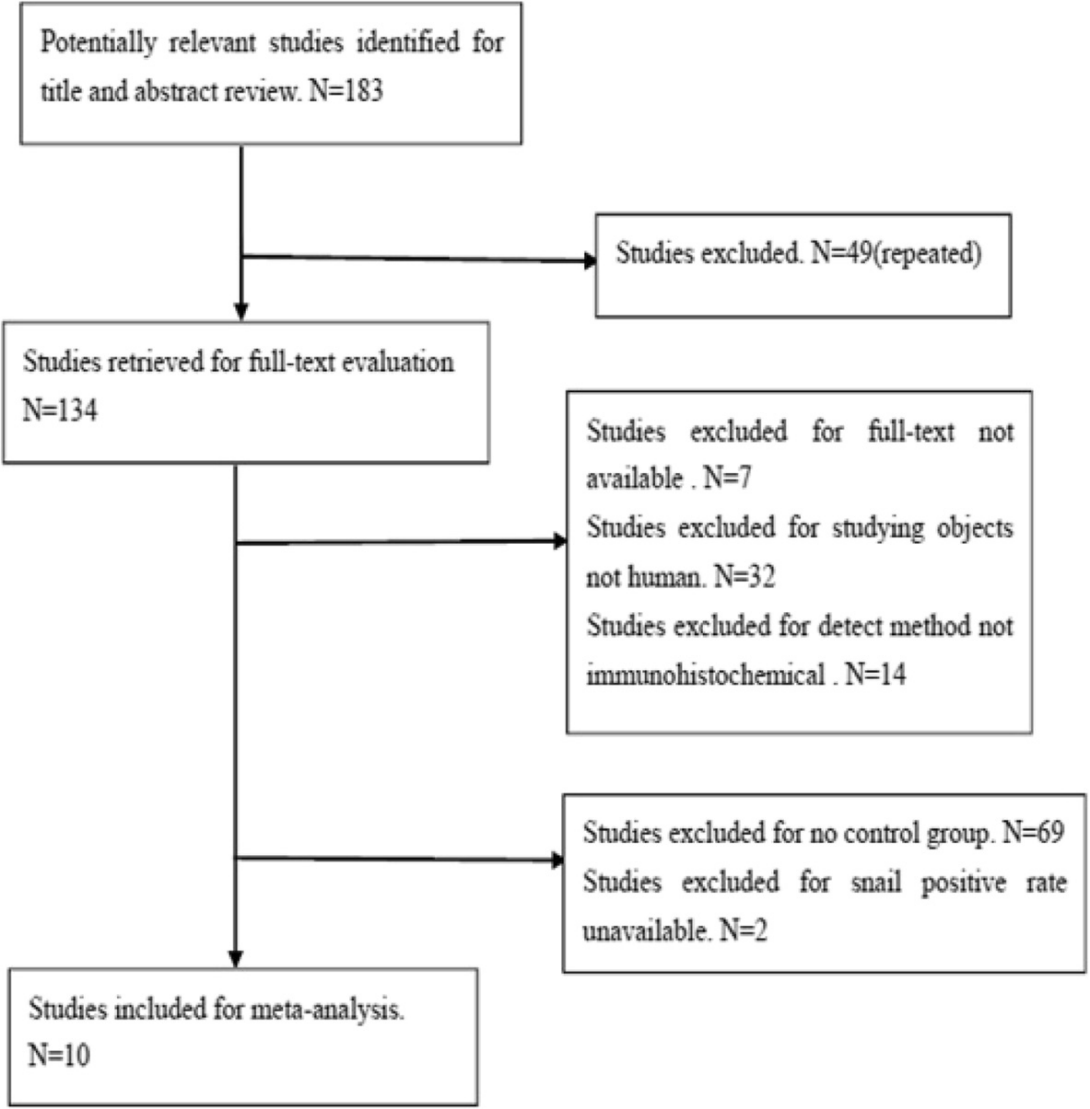
Studies identified with criteria for inclusion and exclusion. After reviewing the abstracts and titles of 183 studies, 173 of them were excluded. In detail, 49 studies were excluded due to repetition; 32 studies were due to non-human subjects; 7 studies were due to non-full-text; 14 studies were due to non-IHC study; 69 studies were due to missing control group; 2 studies were due to missing the snail positive rate. Eventually, 10 articles were collected

After reviewing the abstracts and titles of 183 studies, 173 of them were excluded. In detail, 49 studies were excluded due to repetition; 32 studies were due to non-human subjects; 7 studies were due to non-full-text; 14 studies were due to non-IHC study; 69 studies were due to missing control group; 2 studies were due to missing the snail positive rate. Eventually, 10 articles were collected [16–25] (Fig. 1). Detailed characteristics of these 10 eligible studies are summarized in Table 1. A total of 756 gastric cancer tissue samples, 346 para-carcinoma tissue samples, and 171 normal tissue samples were used in these 10 studies. Eight of them reported the relationship between the snail expression and clinical pathology, enrolled the degree of differentiation, the lymph node metastasis, TNM stage, and invasion depth.

**Table 1:** Main characteristics of the studies included in this meta-analysis

First author	Year	Positive rate											
		Cancer tissue	Adjacent tissue	Normal tissue	Low differentiation	Highly + moderate	TI + TII	TIII + TIV	Superficial	Deep	No metastasis	Metastasis	Quality
Yingfeng Zhu	2007	80/96	33/80	–	56/62	24/34	21/29	59/67	10/16	70/80	23/32	57/64	D
Zhifeng Tang	2010	159/189	26/54	6/32	82/100	61/89	29/46	114/143	–	–	49/73	94/116	D
Yaqin Hao	2011	41/54	22/54	9/30	20/22	21/32	–	–	4/9	17/19	5/11	16/17	D
Shengxi Wang	2011	92/112	28/79	–	66/75	26/37	–	–	15/23	77/89	29/42	63/70	E
Li Jin	2011	78/87	–	7/24	–	–	–	–	–	–	–	–	E
Lina Wang	2011	32/60	16/60	3/20	26/42	6/18	–	–	2/9	30/51	4/7	28/53	D
Wude Zhang	2012	41/48	15/48	–	32/34	9/14	23/27	18/21	22/29	19/19	24/30	7/18	E
Xiaoli Cao	2013	32/45	5/20	–	24/27	8/18	11/20	21/25	9/19	23/26	10/20	22/25	E
Qianjun Li	2013	38/65	–	0/65	–	–	–	–	–	–	–	–	D
Limin Liu	2014	57/80	24/80	–	–	–	6/14	51/66	6/15	51/65	11/24	46/56	C

### Stratification analysis

**Fig. 2:**
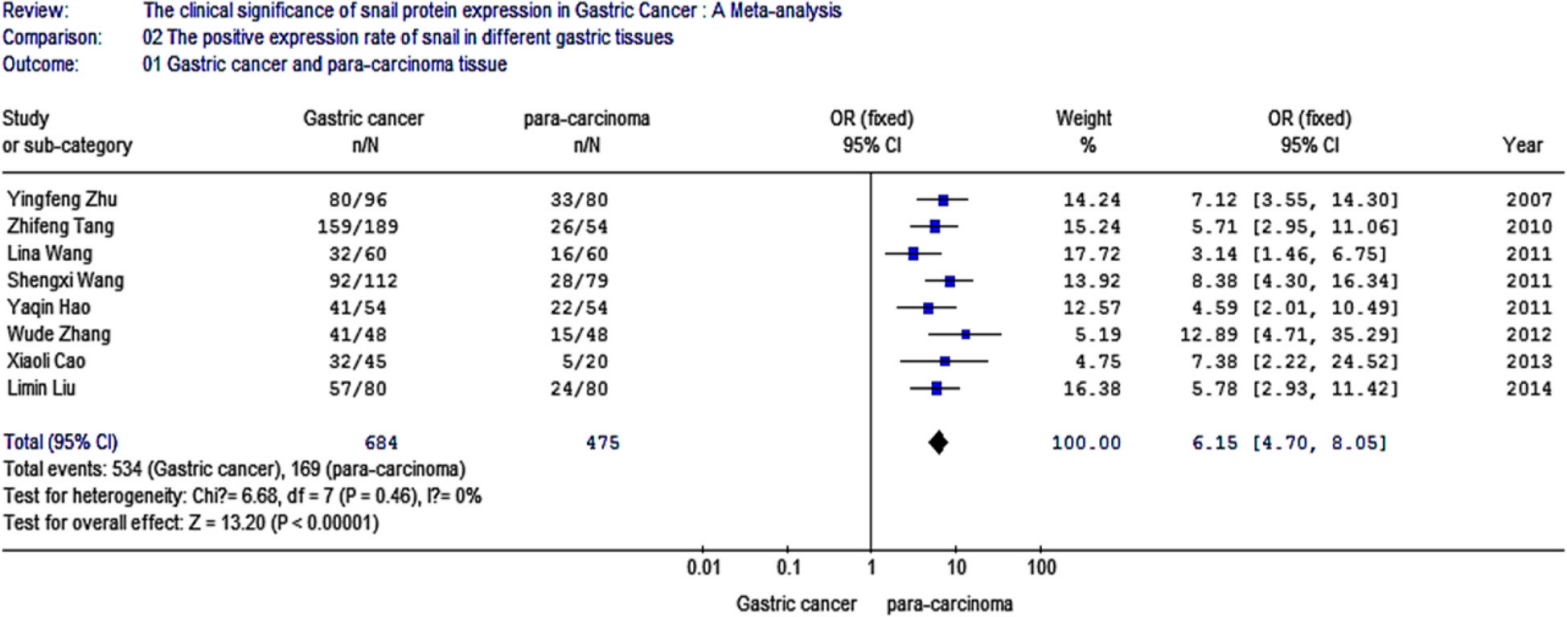
Meta-analysis for the expression of snail protein in gastric cancer and para-carcinoma. Eight of the ten studies compared the expression of snail protein in gastric cancer tissues and the adjacent tissues, including 684 gastric cancer samples and 475 para-carcinoma samples. The I2 value was 0 % and less than 50 %; thus, we chose fixed-effect Mantel-Haenszel model for further analysis. The overall effect was *Z* = 13.20. The odds ratio (OR) was 6.15 with 95 % CI = (4.70, 8.05), and *P* < 0.001

Eight of the ten studies compared the expression of snail protein in gastric cancer tissues and the adjacent tissues, including 684 gastric cancer samples and 475 para-carcinoma samples. The I2 value was 0 % and less than 50 %; thus, we chose fixed-effect Mantel-Haenszel model for further analysis. The overall effect was *Z* = 13.20. The odds ratio (OR) was 6.15 with 95 % CI = (4.70, 8.05), and *P* < 0.001 (Fig. 2).

**Fig. 3:**
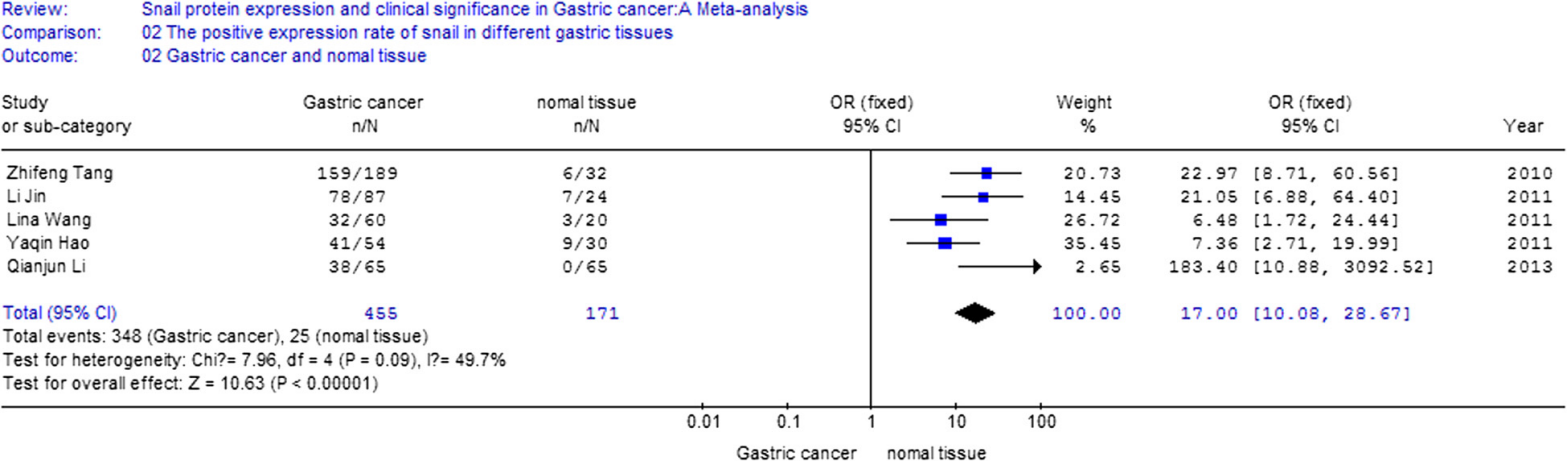
Meta-analysis for the expression of snail protein in gastric cancer and normal tissue. Five of the ten studies compared the positive expression of snail protein in gastric cancer tissues with that in normal tissues, including 455 gastric cancer tissue samples and 171 normal samples. The I2 value was 49.7 % and less than 50 %; thus, we chose fixed-effect Mantel-Haenszel model for further analysis. The overall effect was *Z* = 10.63, OR = 17, 95 % CI = (10.08, 28.67), and *P* < 0.001

Five of the ten studies compared the positive expression of snail protein in gastric cancer tissues with that in normal tissues, including 455 gastric cancer tissue samples and 171 normal samples. The I2 value was 49.7 % and less than 50 %; thus, we chose fixed-effect Mantel-Haenszel model for further analysis. The overall effect was *Z* = 10.63. The odds ratio was 17 with 95 % CI = (10.08, 28.67), and *P* < 0.001 (Fig. 3).

### The relationship between the expression of snail protein and the characteristics of clinical pathology

**Fig. 4:**
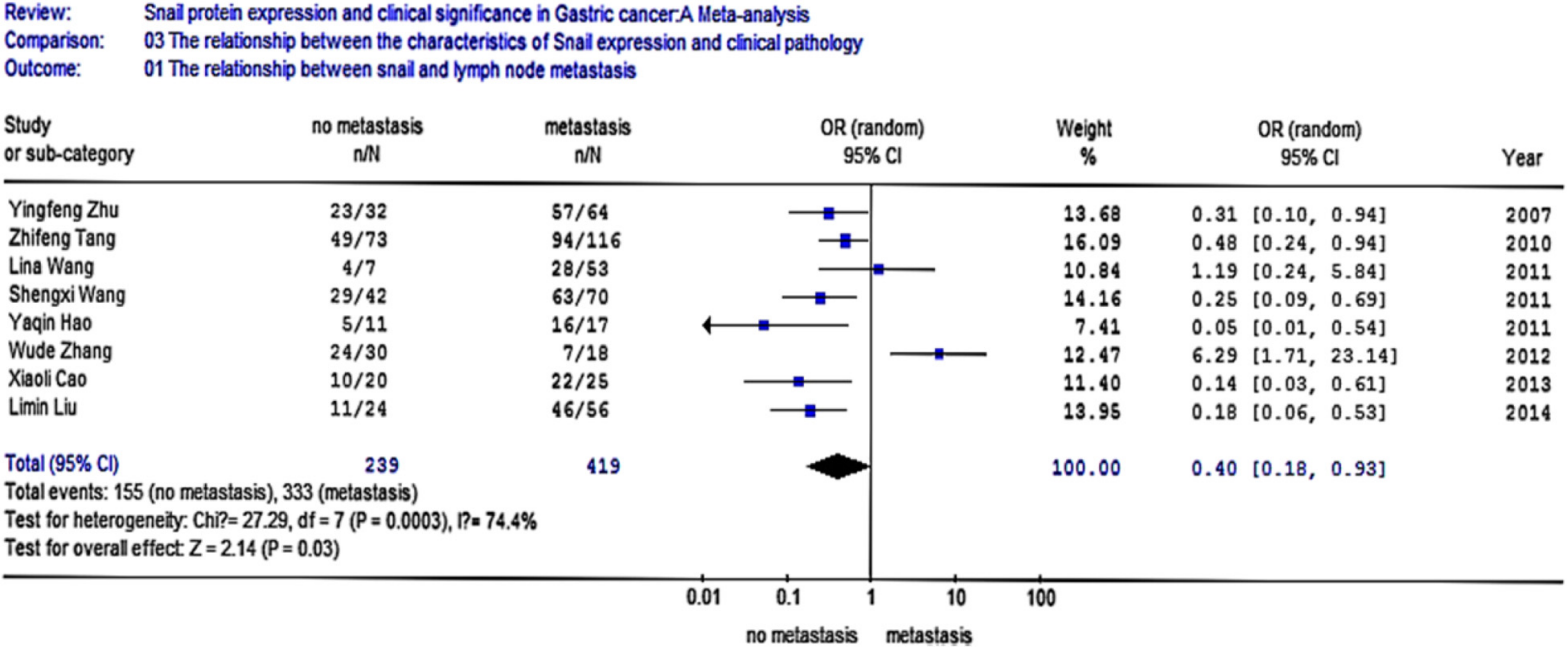
Meta-analysis for the relationship between snail expression and lymph node metastasis. Eight studies analyzed the relationship between snail expression and lymph node metastasis. The results indicated that the I2 value was 74.4 % and greater than 50 %; thus, we chose random-effect model for further analysis. The overall effect was *Z* = 2.14, OR = 0.40, 95 % CI = (0.18, 0.93), and *P* < 0.001

**Fig. 5:**
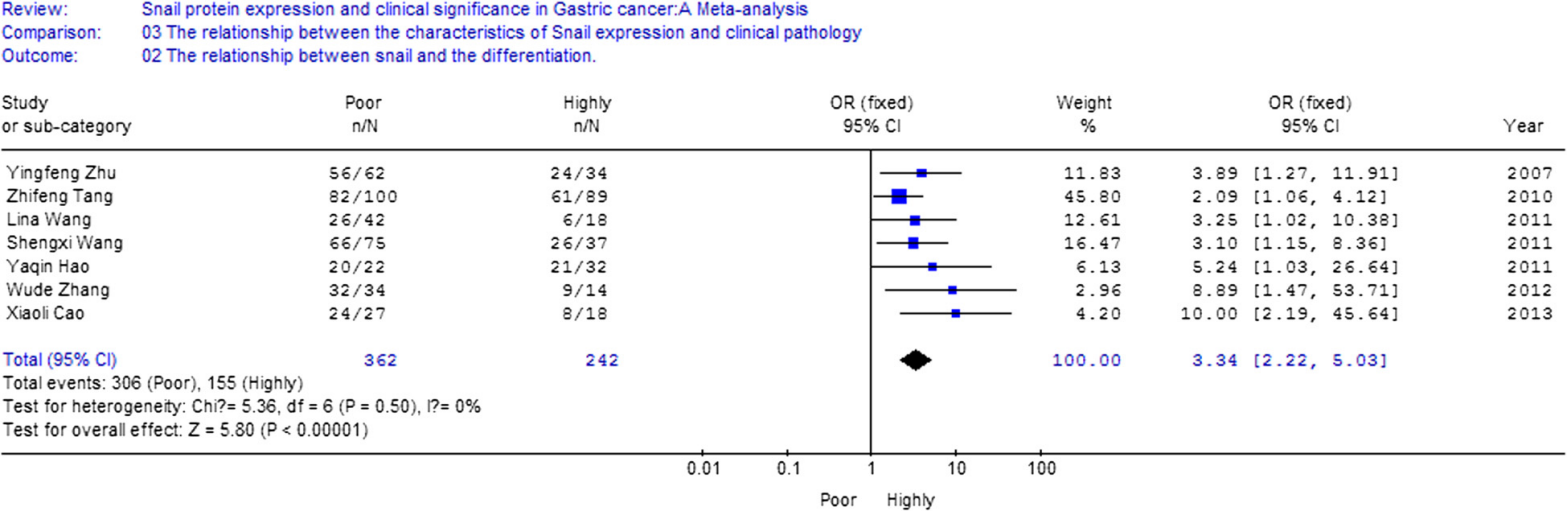
Meta-analysis for the relationship between snail expression and the differentiation. Seven studies analyzed the relationship between snail expression and the differentiation. The result indicated that the I2 value was 0 % and less than 50 %; thus, we chose fixed-effect Mantel-Haenszel model for further analysis. The overall effect was *Z* = 5.80, OR = 3.34, 95 % CI = (2.22, 5.03), and *P* < 0.001

**Fig. 6:**
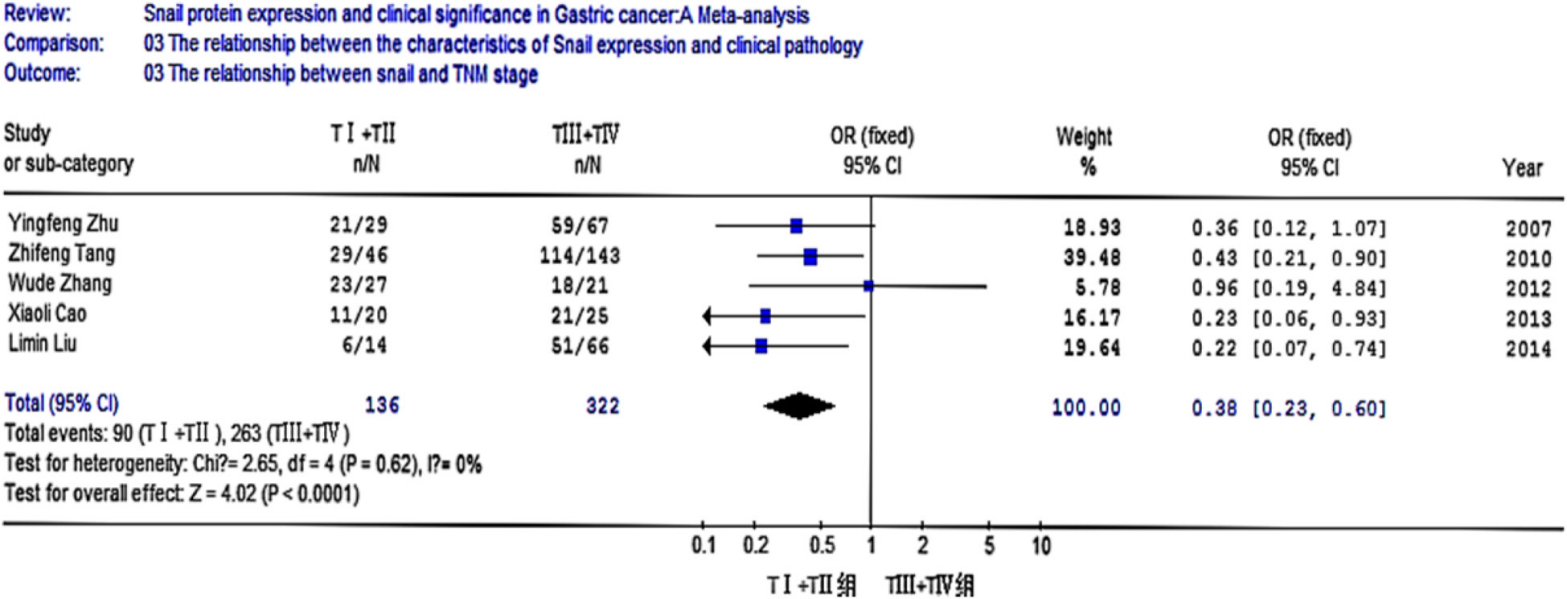
Meta-analysis for the relationship between snail expression and TNM stage. Five studies analyzed the relationship between snail expression and the TNM stage. The result showed that the I2 value was 0 % and less than 50 %, thus we chose fixed-effect Mantel-Haenszel model for further analysis. The overall effect was *Z* = 4.02, OR = 0.38, 95 % CI = (0.23, 0.60), and *P* < 0.001

**Fig. 7:**
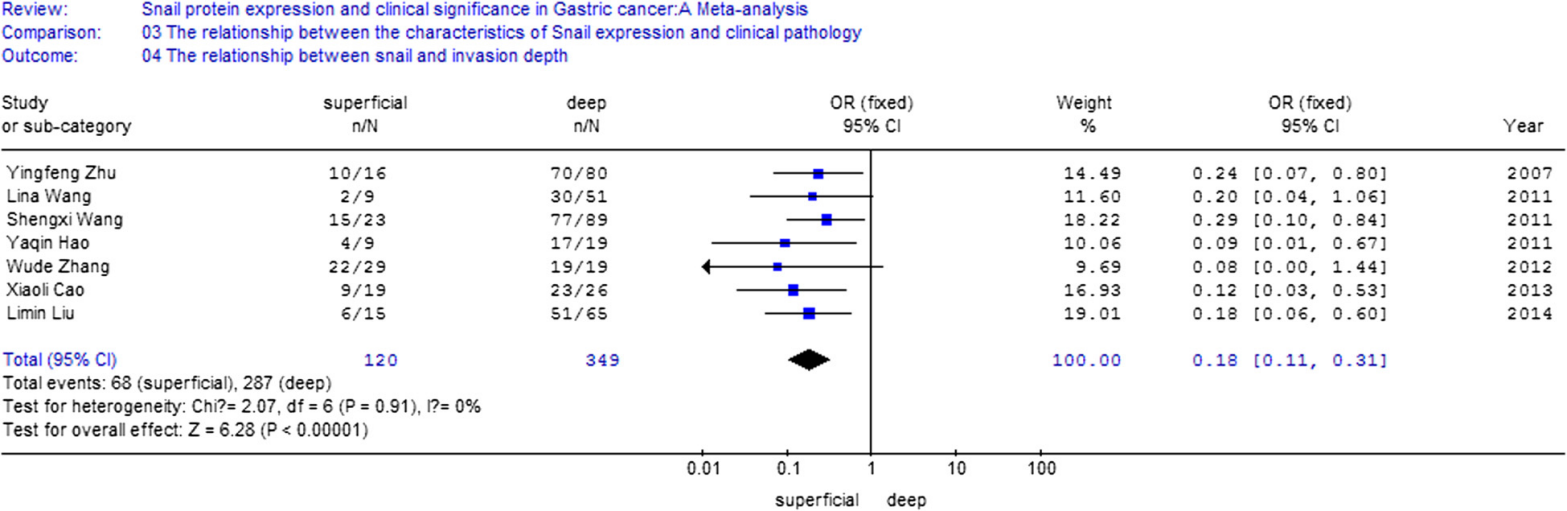
Meta-analysis for the relationship between snail expression and invasion depth. Seven studies analyzed the relationship between snail expression and invasion depth. The result showed that I2 value was 0 % and less than 50 %; thus, we chose fixed-effect Mantel-Haenszel model for further analysis. The overall effect was *Z* = 6.28, OR = 0.18, 95 % CI = (0.11, 0.31), and *P* < 0.001

Eight studies analyzed the relationship between snail expression and lymph node metastasis. The results indicated that the I2 value was 74.4 % and greater than 50 %; thus, we chose random-effect model for further analysis. The overall effect was *Z* = 2.14, OR = 0.40, 95 % CI = (0.18, 0.93), and *P* < 0.001 (Fig. 4). Seven studies analyzed the relationship between snail expression and the differentiation. The result indicated that the I2 value was 0 % and less than 50 %; thus, we chose fixed-effect Mantel-Haenszel model for further analysis. The overall effect was *Z* = 5.80, OR = 3.34, 95 % CI = (2.22, 5.03), and *P* < 0.001 (Fig. 5). Five studies analyzed the relationship between snail expression and the TNM stage. The result showed that the I2 value was 0 % and less than 50 %; thus, we chose fixed-effect Mantel-Haenszel model for further analysis. The overall effect was *Z* = 4.02, OR = 0.38, 95 % CI = (0.23, 0.60), and *P* < 0.001 (Fig. 6). Seven studies analyzed the relationship between snail expression and invasion depth. The result showed that I2 value was 0 % and less than 50 %; thus, we chose fixed-effect Mantel-Haenszel model for further analysis. The overall effect was *Z* = 6.28, OR = 0.18, 95 % CI = (0.11, 0.31), and *P* < 0.001 (Fig. 7).

### Publication bias analysis

**Fig. 8:**
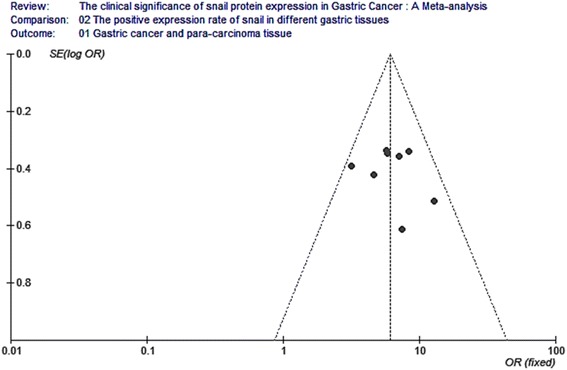
Funnel plot analysis for the expression of snail protein in gastric cancer and para-carcinoma. Funnel plot analysis for publication bias indicated a low likelihood of publication bias

**Fig. 9:**
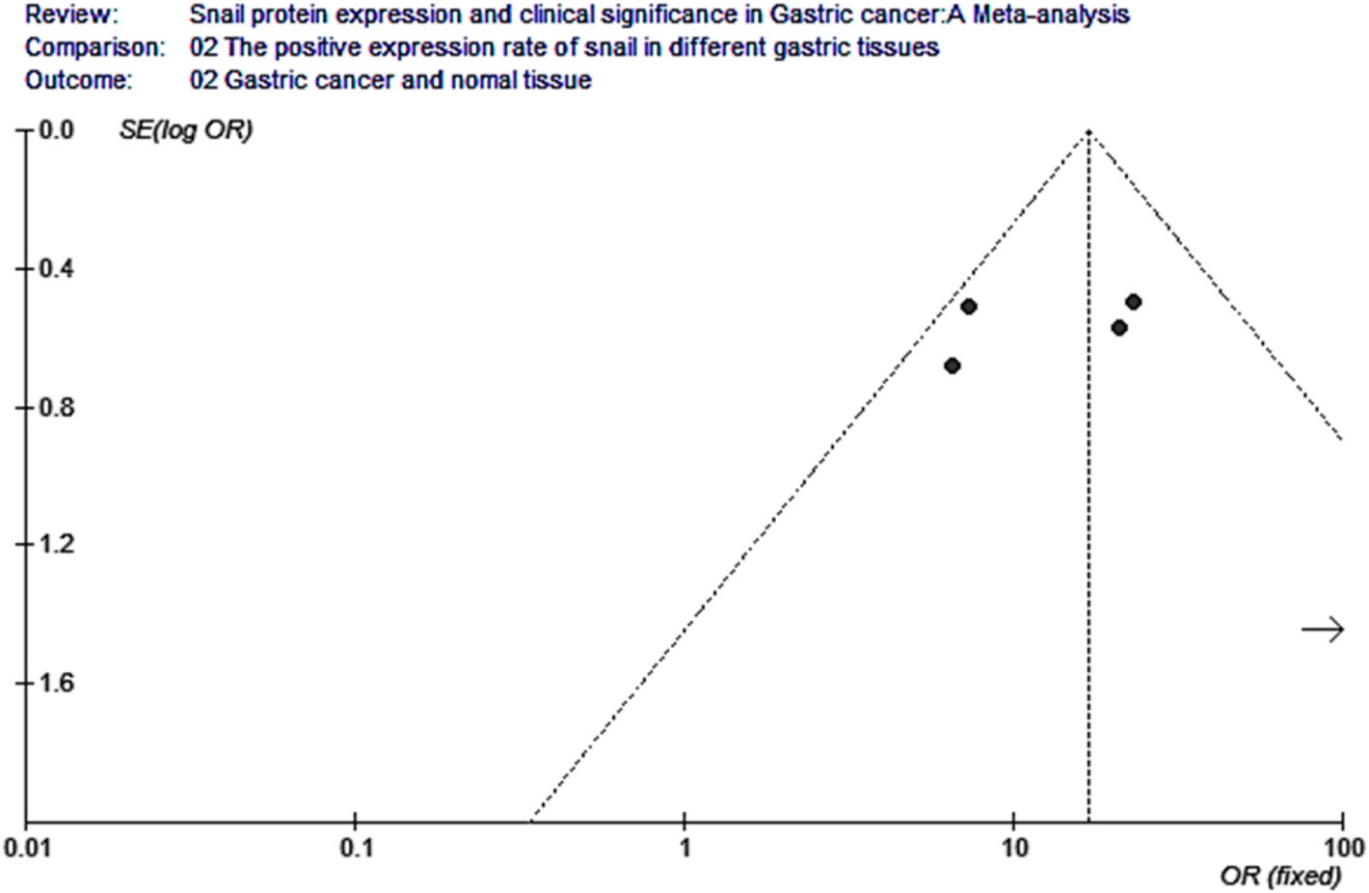
Funnel plot analysis for the expression of snail protein in gastric cancer and normal tissue. Funnel plot analysis for publication bias indicated a low likelihood of publication bias

**Fig. 10:**
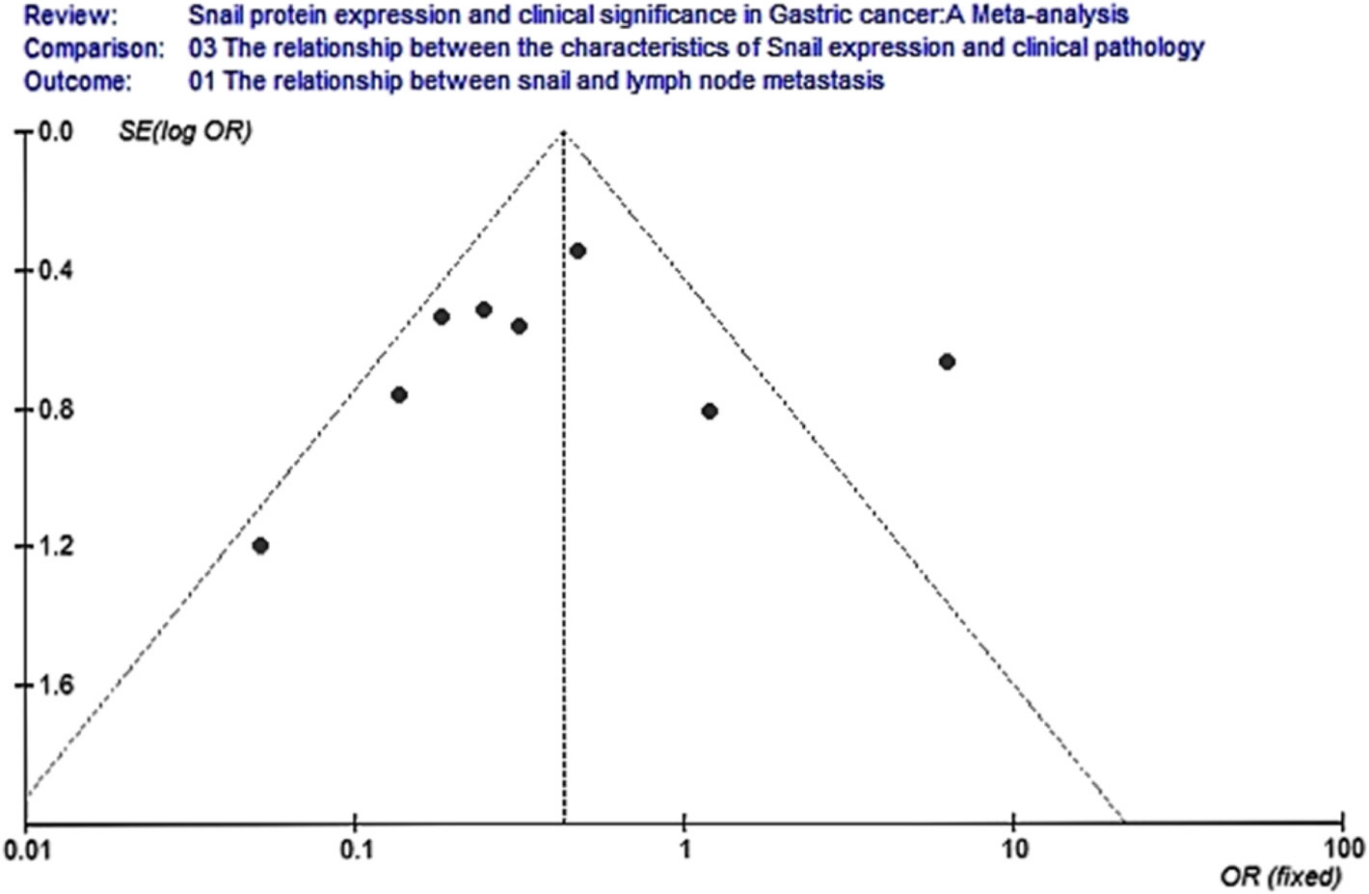
Funnel plot analysis for the relationship between snail expression and lymph node metastasis. Funnel plot analysis for publication bias indicated a low likelihood of publication bias

**Fig. 11:**
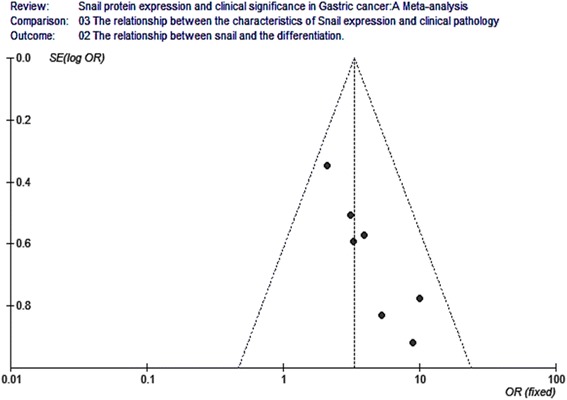
Funnel plot analysis for the relationship between snail expression and the differentiation. Funnel plot analysis for publication bias indicated a low likelihood of publication bias

**Fig. 12:**
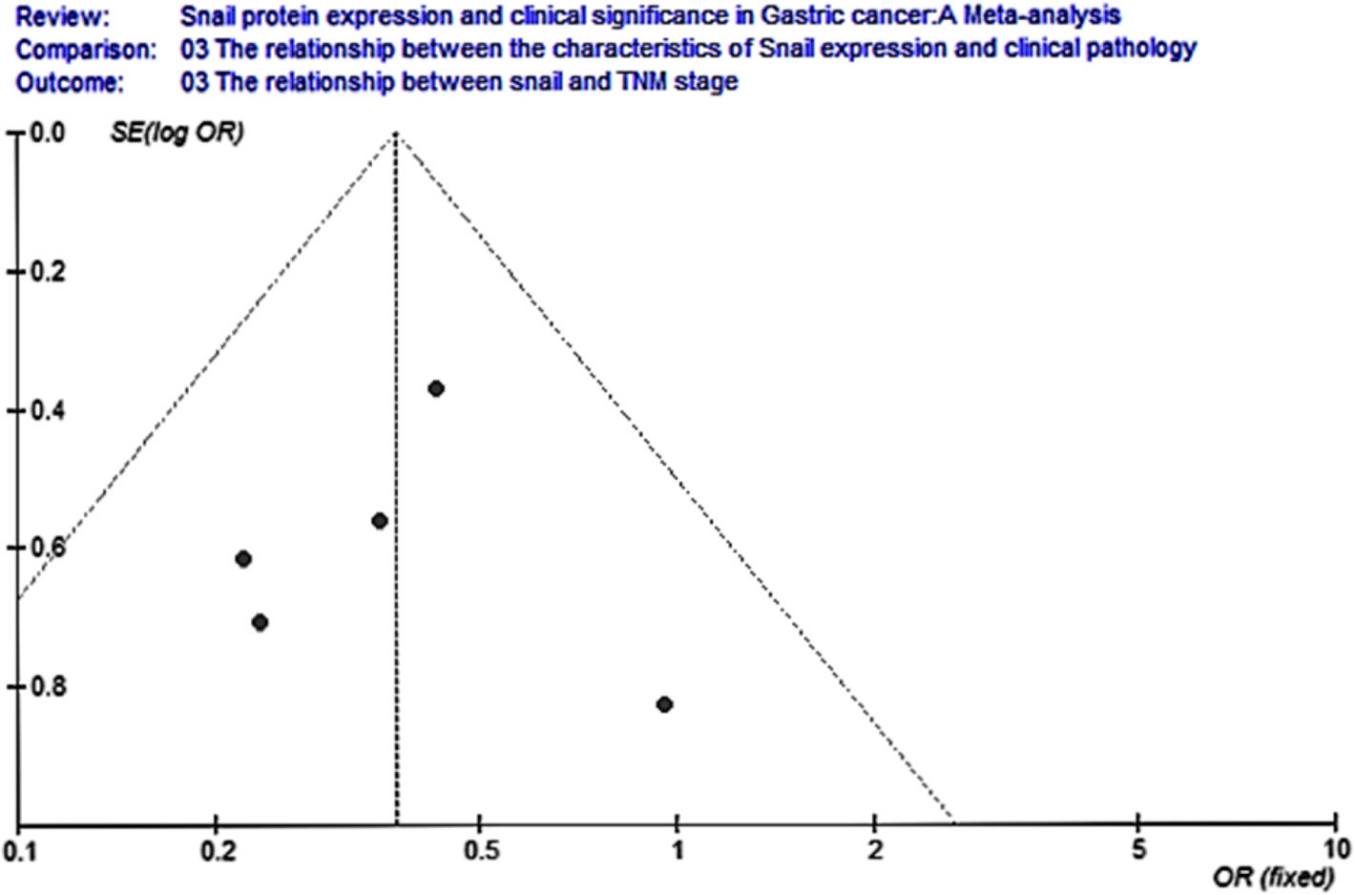
Funnel plot analysis for the relationship between snail expression and the TNM stage. Funnel plot analysis for publication bias indicated a low likelihood of publication bias

**Fig. 13:**
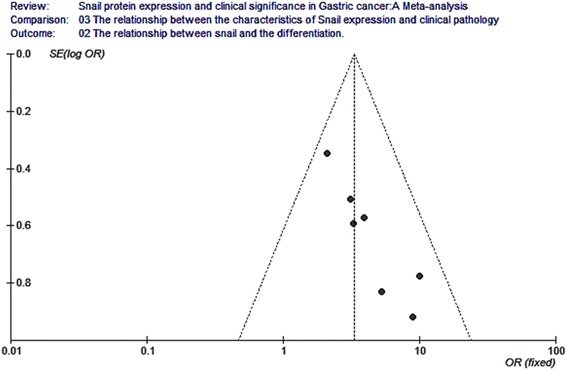
Funnel plot analysis for the relationship between snail expression and the invasion depth. Funnel plot analysis for publication bias indicated a low likelihood of publication bias

Funnel plot analysis for publication bias of these analytical studies (as shown in Figs. 8, 9, 10, 11, 12, and 13) indicated a low likelihood of publication bias.

## Discussion and conclusions

**Fig. 14:**
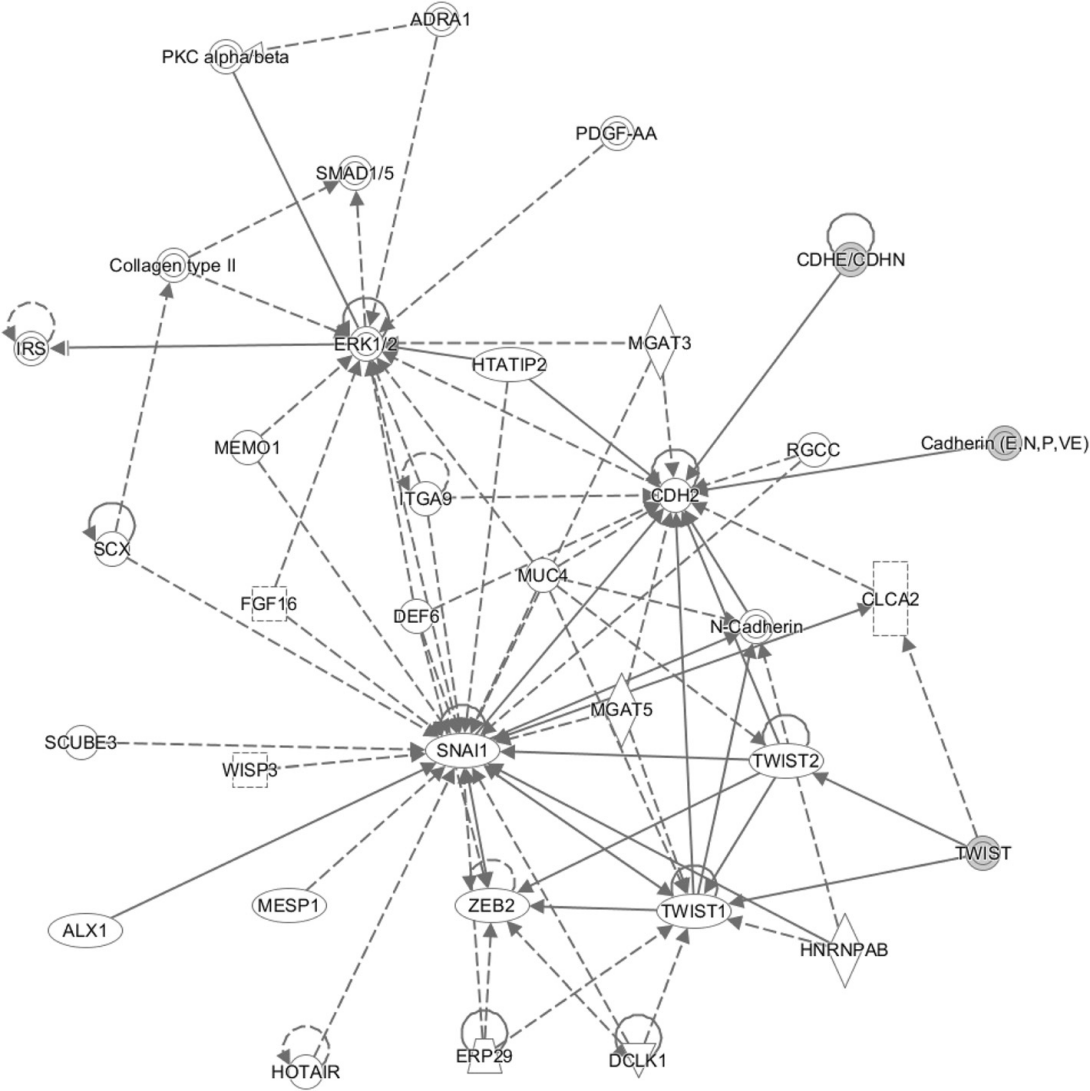
Gene network analysis. The target genes of cancer-induced differentially expressed protein were used to run the IPA tool for gene network analysis. These genes around *triangles* highlighted genes that are involved in immunity system development function. The network score described in the “Methods” section for the network is 39. The *solid lines* connecting the molecules here represent a direct relation and *dotted lines* an indirect relation

The emerging roles of some key, EMT-related proteins in cancer progression and their close relationship with clinical pathology parameters make them attractive for developing diagnostic biomarkers and therapies [26]. The transcriptional repression of E-cadherin is mediated mainly by zinc finger transcription factors related to the snail family (SNAIL1), zinc finger E-box binding homeobox-2 (ZEB2), and basic helix-loop-helix family (TWIST) [27, 28]. Network analysis (Fig. 14) revealed that snail expression was significantly correlated with the expression of ZEB2, TWIST (Twist1 and Twist2), and N-cadherin (CDH2). These gene expressions may be regulated by snail at transcriptional level, and they also interact with each other. N-cadherin, encoded by the CDH2 gene, mediates cell-cell adhesion and renders tumor cell migration and invasion [29]. N-cadherin was reported to be a prognostic marker [30], and the up-regulation correlated with advanced TNM stage and poor survival [31]. In addition, TWIST can modulate N-cadherin expression through directly interacting with an E-box, a regulatory element within intron 1 of CDH2 [32], and expression of TWIST appears to be indispensable for the entry of tumor cells into the bloodstream, a significant early step towards metastasis [33]. ZEB2 is also known as SIP1, which interacts through its COOH-terminal region with E-box element of E-cadherin gene promoter and mediates its transcriptional repression by recruiting corepressor complexes [34, 35]. These transcription factors form signaling networks that could initiate and sustain the mesenchymal phenotypes of tumor cells; therefore, the expression of these proteins could define EMT occurrence in a tumor setting. For example, a study in primary human gastric cancers revealed elevated snail and twist expressions in diffuse-type gastric cancer, whereas ZEB2/SIP1 was primarily expressed in the intestinal type [36] (Fig. 14).

This meta-analysis was aimed to examine the expression of transcription factor snail in different tissue samples and the relationship between increased snail expression and clinicopathological features of gastric cancer. This study combined 756 gastric cancer tissue samples, 346 para-carcinoma samples, and 171 normal tissue samples from 10 individual studies. The results indicated that snail expression is higher in gastric cancer tissues than that in para-carcinoma tissues and normal tissues, respectively (OR = 6.15, 95 % CI = 4.70, 8.05; OR = 17, 95 % CI = 10.08, 28.67). Furthermore, closed correlations were observed between snail expression and clinicopathological characteristics that included the lymph node metastasis, the degree of differentiation, TNM stage, and invasion depth. The positive expression rate of snail was higher in gastric cancer tissues with lymphatic metastasis, OR = 0.40, 95 % CI = (0.18, 0.93). The higher positive rate of snail is connected with the lower differentiation degree, OR = 3.34, 95 % CI = (2.22, 5.03). The positive expression of snail was higher at late clinical stage, OR = 0.38, 95 % CI = (0.23, 0.60). Moreover, it appeared that the deeper the infiltration was, the higher the expression of snail was, OR = 0.18, 95 % CI = (0.11, 0.31).

The result of funnel plot indicated an imminent possibility of publication bias. Two potential biases might be introduced. First, the languages in collected papers were used in both Chinese and English, which may lead to a language bias. Second, the majority of collected studies did not use blind method, which might result in a measurement bias. Hence, the large-scale samples and double blind statistical tests will be investigated in the future study. Additionally, our review only collected the publications that have full text, since data that can be used for the methodology assessment and meta-analysis were only available in these publications with full text.

Our meta-analysis indicated that snail was highly expressed in gastric cancer. In addition, the overexpression of snail is significantly associated with tumor progression and metastasis.
